# Recovery practice in community mental health teams: national survey

**DOI:** 10.1192/bjp.bp.114.160739

**Published:** 2016-10

**Authors:** M. Leamy, E. Clarke, C. Le Boutillier, V. Bird, R. Choudhury, R. MacPherson, F. Pesola, K. Sabas, J. Williams, P. Williams, M. Slade

**Affiliations:** **M. Leamy**, PhD, **E. Clarke**, MBBS, **C. Le Boutillier**, MSc, **V. Bird**, PhD, Institute of Psychiatry, Psychology & Neuroscience, King's College London, London; **R. Choudhury**, MBBS, **R. MacPherson**, MBBS, ^2^gether NHS Foundation Trust, Gloucester; **F. Pesola**, PhD, **K. Sabas**, BSc, **J. Williams**, PhD, **P. Williams**, MSc, **M. Slade**, PhD, Institute of Psychiatry, Psychology & Neuroscience, King's College London, London, UK

## Abstract

**Background**

There is consensus about the importance of ‘recovery’ in mental health services, but the link between recovery orientation of mental health teams and personal recovery of individuals has been underresearched.

**Aims**

To investigate differences in team leader, clinician and service user perspectives of recovery orientation of community adult mental health teams in England.

**Method**

In six English mental health National Health Service (NHS) trusts, randomly chosen community adult mental health teams were surveyed. A random sample of ten patients, one team leader and a convenience sample of five clinicians were surveyed from each team. All respondents rated the recovery orientation of their team using parallel versions of the Recovery Self Assessment (RSA). In addition, service users also rated their own personal recovery using the Questionnaire about Processes of Recovery (QPR).

**Results**

Team leaders (*n* = 22) rated recovery orientation higher than clinicians (*n* = 109) or patients (*n* = 120) (Wald(2) = 7.0, *P* = 0.03), and both NHS trust and team type influenced RSA ratings. Patient-rated recovery orientation was a predictor of personal recovery (*b* = 0.58, 95% CI 0.31–0.85, *P*<0.001). Team leaders and clinicians with experience of mental illness (39%) or supporting a family member or friend with mental illness (76%) did not differ in their RSA ratings from other team leaders or clinicians.

**Conclusions**

Compared with team leaders, frontline clinicians and service users have less positive views on recovery orientation. Increasing recovery orientation may support personal recovery.

Focusing mental health interventions towards supporting recovery (known as having a ‘recovery orientation’) is national mental health policy in many countries.^1–3^ Recovery-oriented approaches offer a transformative conceptual framework for practice, culture and service delivery in mental health service provision.^4^ Such policy suggests there is an underlying link between the recovery orientation of teams and the experience of recovery. For example, a survey of 67 assertive community treatment teams in Canada using the Recovery Self Assessment (RSA) scale^5^ found that recovery orientation was associated with more positive client outcomes.^6^ The recovery orientation of teams can be assessed from multiple stakeholder perspectives, which may differ, although a USA study found high levels of agreement between directors (managers) and people-in-recovery (service users).^5^ The proportion of the mental health workforce with ‘lived experience’ (personal experience of mental health problems or supporting someone with mental health problems) is unknown, but dual identity as a clinician with lived experience represents a potential resource in the system,^7^ and increasing this proportion is emerging as a target for organisational transformation.^8^ The aim of this study was to investigate differences in team leader, clinician and service user perspectives on recovery orientation of community adult mental health teams in England. Objectives were (a) to compare variations between National Health Service (NHS) trust, team type and participant ratings of recovery orientation of mental health teams; (b) to explore the relationship between service user ratings of recovery orientation and their ratings of personal recovery and the extent to which they assess teams as recovery-oriented; and (c) to test the hypothesis that clinician-rated recovery orientation differs between clinicians with and without lived experience.

## Method

### Design

The study used a cross-sectional survey design. Ethical approval was obtained from South East London Research Ethics Committee 4 (10/H0807/4).

### Sample and setting

The study took place in six NHS mental health trusts in England: Coventry and Warwickshire Partnership NHS Trust, Leicestershire Partnership NHS Trust, ^2^gether NHS Foundation Trust, Devon Partnership NHS Trust, Tees, Esk and Wear Valley NHS Foundation Trust and Leeds Partnership NHS Trust. A purposive sample of NHS trusts was chosen to include a mix of regions (Midlands, South West, North East, North West), levels of urbanisation, socioeconomic deprivation status, ethnic diversity, organisational size and structures (foundation or non-foundation). Teams met the inclusion criteria if they were adult community mental health teams (CMHTs) using the Care Programme Approach (CPA), a framework for coordination of mental health assessment and treatment used by multiprofessional mental health teams following national service models (CMHTs, support and recovery, early intervention, assertive outreach). All team leaders were eligible. Clinicians were eligible if they had direct clinical contact with service users. Service users within the team were eligible for inclusion if they met the following criteria: (a) were aged 18–65 years, (b) had no immediate plans for discharge, (c) spoke and understood English, (d) were able to give consent, and (e) were sufficiently well to participate (in the opinion of the clinician who works most closely with the service user). Individuals were excluded if they were receiving in-patient care.

### Measures

Recovery orientation of mental health teams was measured using the RSA, which has parallel versions for team leaders, clinicians and service users.^5^ Each version contains 36 items rating practices associated with supporting recovery. Example questions include ‘Staff use a language of recovery (i.e. hope, high expectations, respect) in everyday conversations' and ‘Staff help to monitor the progress I am making towards my personal goals on a regular basis’. Participants rate the degree to which their team engaged in the practice on a five-point Likert scale from one (strongly disagree) to five (strongly agree) or not applicable. The RSA can be scored as a total sum score ranging from 36 (low recovery orientation) to 180 (high recovery orientation; alpha (α) = 0.94) or as five subscales: (a) life goals *v.* symptom management (α = 0.88), (b) user involvement and recovery education (α = 0.84), (c) diversity of treatment options (α = 0.72), (d) rights and respect (α = 0.61), and (e) individually tailored services (α = 0.64). Mean RSA scores were used for subscales.

The Questionnaire about the Processes of Recovery (QPR) is a 22-item self-report outcome measure of personal recovery, completed only by service users.^9^ They are asked to rate their own progress towards personal recovery.^9^ Example questions include ‘I feel that my life has a purpose’ and ‘I can recognise the positive things I have done’. Each item comprises a pro-recovery statement rated from one (low recovery) to five. We calculated the QPR score following recent guidelines from the developers who suggest using 15 of the original 22 items to extrapolate a recovery score (α = 0.95).^10^

In the survey, clinicians were also asked ‘Do you have experience of supporting a family member or friend with mental health problems?’, ‘Have you ever experienced mental health problems?’, ‘Have you ever used mental health services?’ (all yes/no responses) and ‘Have you disclosed this information to your work colleagues?’ (responses of yes, I am fully open with my colleagues when appropriate; not fully, but I have disclosed in confidence to at least one of my colleagues; or no).

### Procedure

In each of these six participating sites, four teams were randomly selected using a random number generator (www.randomisation.com). One NHS trust dropped out, so we recruited an additional four teams from the remaining NHS trusts. The team leader was approached and asked for consent for their team to participate, with alternative teams approached, according to randomisation order, if necessary. In each team a convenience sample of five clinicians were identified, in liaison with the team leader, with alternatives approached where necessary. Also, ten service users were randomly chosen using the random number generator from an anonymised case-load list supplied by the team leader. Clinicians then asked these randomised service users whether they would be willing to be contacted by the research team. If the service user refused, or was ineligible, then the next service user on the list was selected. Surveys for each participant group – team leader (RSA), clinician (RSA) and service user (RSA and QPR) – were offered in multiple forms (post, email or telephone). All participants were asked to either post or email responses direct to the research team anonymously, or a telephone interview was arranged with a researcher if preferred. In the four settings where there was sufficient research team capacity, the service user group were also offered face-to-face interviews, which included assistance where requested. Service users were paid by £10 gift voucher, sent in advance of receipt of completed questionnaire (as this increases response rate^11^). Survey data were collected between September 2010 and August 2012.

The data-set was validated by checking for missing data and outliers (although no outliers were found), with items checked against the original questionnaires to minimise transcription errors. Missing data were imputed using mean replacement following the authors' guidelines for the RSA measures when less than 20% of data were missing on the QPR scale. Participants with more than 20% of missing data were excluded from the analysis.

### Analysis

Regression analyses were conducted by entering the predictors into the model for each objective (objective 1: respondent type; objective 2: QPR score; objective 3: personal experience) with NHS trust entered as a covariate. Sensitivity analysis adjusted the model for covariates: age, gender, ethnicity (White *v.* Black and minority ethnic), time using mental health services (service user) or length of NHS employment (clinician). We used random effects regression models with maximum likelihood estimation using the ‘xtmixed’ command in Stata 11 to account for clustering at the team level as respondents in the same team might not be independent. Bonferroni correction was used to adjust for multiple testing.

## Results

Seven NHS trusts were approached, of whom six (86%) participated and one did not respond. Four additional teams were recruited from remaining NHS trusts to replace the four teams from the non-participating NHS trust, so 28 (100% of target) teams participated, comprising nine psychosis-specific CMHTs, eight community mental health teams, four support and recovery teams, four early intervention teams and three assertive outreach teams.

**Fig. 1:**
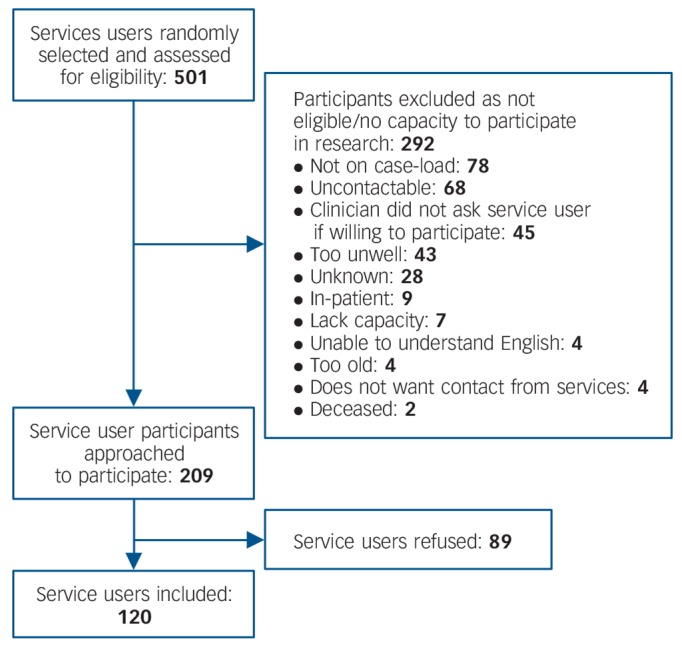
Flow diagram for service user recruitment.

A total of 22 (79%) of 28 team leaders and 109 (78% of 140 target) clinicians participated, comprising nurses (*n* = 58, 44%), social workers (*n* = 25, 19%), support and recovery workers (*n* = 16, 12%), occupational therapists (*n* = 14, 11%), psychiatrists (*n* = 5, 4%), psychologists (*n* = 5, 4%) and other/missing (*n* = 6, 5%). The majority were women (*n* = 90, 69%) and White British (*n* = 119, 91%), with a mean age of 45.0 years (s.d. = 8.7), a mean time in current post of 6.0 years (s.d. = 5.2), and a mean time working in mental health services of 16.6 years (s.d. = 9.7). A total of 120 (43%) of the target 280 service users were recruited, as shown in Fig. 1. The characteristics of service user group are shown in Table 1.

**Table 1:** Service user characteristics (*n* = 120)

Service user characteristics	*n* (%)
Gender	
Male	72 (60)
Female	48 (40)

Ethnicity	
White British	97 (81)
White Other	4 (3)
Asian/Asian British–Pakistani	4 (3)
Mixed White and Asian	3 (3)
Mixed White and Black–Caribbean	2 (2)
Asian/Asian British–Indian	2 (2)
Black/Black British–Caribbean	2 (2)
Black/Black British–African	1 (1)
Other	1 (1)
Missing data	4 (3)

Self-reported diagnosis	
Mood disorder	40 (33)
Psychotic disorder	29 (24)
Anxiety disorder	7 (6)
Personality disorder	6 (5)
Other	2 (2)
Missing data	36 (30)

Responses from the clinician and team leader groups were primarily by post (*n* = 97, 74%), with face-face interviews (*n* = 20, 15%), email (*n* = 12, 9%) and phone interview (*n* = 2, 2%) also used. Responses from the service user group were by post (*n* = 62, 52%), face-face interviews (*n* = 34, 28%) and phone (*n* = 24, 20%). Following pro-rating there was complete information for 239 (out of 251) participants on the RSA and covariates, recruited across 28 teams. Service users were more likely to have missing data and therefore be excluded than clinicians and team leaders (8% *v.* 2% clinicians/team leaders; χ^2^(1) = 6.4, *P* = 0.012). Excluded people did not differ from those included on age, gender, time in NHS, diagnosis or ethnicity. The final sample comprised 108 clinicians, 21 team leaders and 110 service users (who also had complete information on the QPR).

### NHS trust, team and participant variations on RSA

Analyses were conducted on 239 respondents across 28 teams with a mean of 9 observations per team cluster (range 1 to 16). There was an effect of clustering at team-level (χ^2^(2) = 4.7, *P* = 0.015, intraclass correlation (ICC) = 9%). We therefore checked whether variability across teams was explained by NHS trust (for example, because of distinctive organisational cultures). NHS trust was entered as a predictor in the null multilevel model, and clustering at team level was weakened (χ^2^(1) = 2.2, *P* = 0.071, ICC = 6%) and a 23% reduction of unexplained variance on the RSA measure across teams was observed. These results indicate that NHS trust explained some of the variation across teams.

We then investigated whether some types of team were rated as more recovery-oriented than others. The results showed that overall RSA scores varied across team types (Wald(4) = 22.14, *P*<0.001). We ran pairwise comparisons between all team types (see Table 2). After adjusting for multiple testing, RSA scores were higher among early intervention teams than support and recovery teams (*b* = −0.39, 95% CI −0.61 to −0.17, *P* = 0.001) and community and mental health teams (*b* = −0.67, 95% CI −1.08 to −0.26, *P* = 0.001).

**Table 2:** Comparisons between team types (*n* = 28)

	Pairwise comparisons^a^	
Team type	*b* (95% CI)	*P*
Early intervention *v.* assertive	−0.23 (−0.51 to 0.05)	0.103

Early intervention *v.* support and recovery	**−0.39 (−0.61 to −0.17)**	**0.001**

Early intervention *v.* psychosis	−0.41 (−0.78 to −0.05)	0.025

Early intervention *v.* community mental health team	**−0.67 (−1.08 to −0.26)**	**0.001**

Assertive *v.* support and recovery	−0.16 (−0.48 to 0.15)	0.306

Assertive *v.* psychosis	−0.19 (−0.50 to 0.13)	0.243

Assertive *v.* community mental health team	−0.44 (−0.82 to −0.07)	0.018

Support and recovery *v.* psychosis	−0.02 (−0.43 to 0.38)	0.907

Support and recovery *v.* community mental health team	−0.28 (−0.73 to 0.17)	0.217

Psychosis *v.* community mental health team	−0.26 (−0.45 to −0.06)	0.010

a. Significant findings are in bold.

Third, we investigated variability in the RSA mean scores for the three participant groups shown in Table 3. Regression of respondent group on mean RSA scores showed that the participant groups differed on their RSA scores (Wald(2) = 7.0, *P* = 0.031), with the team leader group responses higher than the clinician group (*b* = −0.30, 95% CI −0.53 to −0.08, *P* = 0.008) and the service user group (*b* = −0.25, 95% CI −0.48 to −0.03, *P* = 0.029), although the latter difference became non-significant after adjustment. We found no difference between the clinician and service user group (*b* = −0.05, 95% CI −0.18 to 0.08, *P* = 0.432). The effect of participant type on RSA was still present after including the covariates (Wald χ^2^(2) = 9.4, *P* = 0.009). None of the covariates was found to be a predictor of RSA mean scores.

**Table 3:** Recovery Self Assessment (RSA) scores by respondent group

	RSA subscale, mean (s.e.)					
Respondent group	Life goals *v.* symptom management	User involvement and recovery education	Diversity of treatment options	Rights and respect	Individually tailored services	RSA total
Clinician	4.00 (0.05)	2.95 (0.07)	3.25 (0.07)	4.05 (0.06)	3.56 (0.06)	3.59 (0.05)

Team leader	4.31 (0.12)	3.21 (0.13)	3.47 (0.15)	4.45 (0.12)	4.10 (0.12)	3.90 (0.11)

Service user	3.81 (0.07)	3.31 (0.08)	3.45 (0.08)	3.91 (0.07)	3.60 (0.07)	3.63 (0.06)

Fourth, we looked for variability in the RSA subscales for the three types of participant. In total, 202 of the 239 participants had information on all five subscales and were included in the analyses. There was a mean of eight observations per cluster. We compared group scoring across the RSA scales by running a regression analysis of RSA scores on respondent type with random intercept for clustering at the level of team with the model adjusted for NHS trust and covariates. Overall Wald test and pairwise comparisons are shown in Table 4. A Bonferroni correction was used to adjust for multiple testing. The overall Wald test showed that there was an effect of participant type on four of the five subscales. The service user group's ratings for subscale 2 (user involvement and recovery education) were higher than the clinician group's, but the main overall difference was that the team leader group rated a higher recovery orientation on subscales 3, 4 and 5 (diversity of treatment options, rights and respect, individually tailored services) than both the service user and clinician groups.

**Table 4:** Overall Wald test and pairwise comparisons^a^

	1. Life goals *v.* symptom management			2. User involvement and recovery education			3. Diversity of treatment options			4. Rights and respect			5. Individually tailored services		
RSA subscale	*b* (95% CI)	Wald (2)	*P*	*b* (95% CI)	Wald (2)	*P*	*b* (95% CI)	Wald (2)	*P*	*b* (95% CI)	Wald (2)	*P*	*b* (95% CI)	Wald (2)	*P*
Overall Wald		**18.3**	**<0.001**		**20.1**	**<0.001**		3.5	0.174		**17.4**	**<0.001**		**20.3**	**<0.001**

Pairwise comparison, *z*															
Team leader *v.* clinician	**−0.38**		**0.003**	−0.28		0.040	−0.22		0.179	**−0.46**		**0.001**	**−0.59**		**<0.001**
	**(−0.63 to −0.13)**			(−0.55 to −0.01)			(−0.53 to 0.10)			**(−0.72 to −0.19)**			**(−0.85 to −0.33)**		
Team leader *v.* service user	**−0.57**		**<0.001**	0.11		0.452	−0.05		0.761	**−0.59**		**<0.001**	**−0.55**		**<0.001**
	**(−0.83 to −0.30)**			(−0.17 to 0.39)			(−0.38 to 0.28)			**(−0.87 to −0.31)**			**(−0.82 to −0.28)**		
Clinician *v.* service user	0.19		0.025	**−0.39**		**<0.001**	−0.17		0.111	0.14		0.123	−0.04		0.608
	(0.02 to 0.35)			**(−0.57 to −0.22)**			(−0.37 to 0.04)			(−0.04 to 0.31)			(−0.21 to 0.13)		

RSA, Recovery Self Assessment.

a. Significant findings after Bonferroni correction shown in bold.

### Recovery support and personal recovery in the service user group

**Fig. 2:**
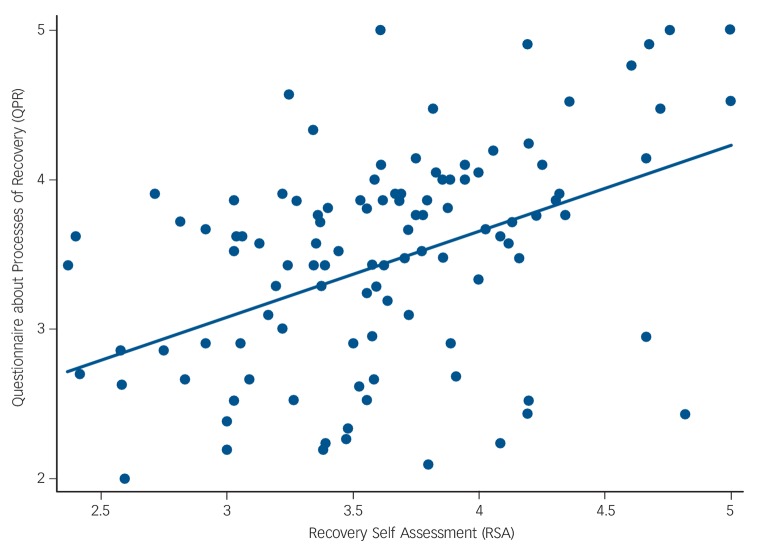
Scatterplot with line of best fit capturing the relationship between Recovery Self Assessment (RSA) and Questionnaire about Processes of Recovery (QPR) scores (*n* = 110).

We investigated whether service user ratings of recovery orientation were significantly associated with personal recovery. Analyses were conducted on 110 (of 120) of the service user group, across 26 teams with a mean of 4 observations per cluster, as these participants had complete information on the RSA, QPR and covariates. The scatterplot in Fig. 2 shows the relationship between RSA scores and QPR scores. The RSA scores were positively associated with QPR total scores (*b* = 0.53, 95% CI 0.32–0.74, *P*<0.001). Adjusting the model for covariates confirmed the results (*b* = 0.58, 95% CI 0.31–0.85, *P*<0.001).

### Recovery support and lived experience

Among the clinician and team leader groups, 100 (76%) reported having experience of supporting a family member or friend and 50 (39%) reported having had personal experience of mental health problems. Of those who reported personal experience, 24 (48%) had fully disclosed this experience to workplace colleagues, 16 (32%) had partially disclosed this and 10 (20%) had not disclosed this. Of the 24 who had fully disclosed their personal experience of mental illness, 19 (79%) reported they had received support and 5 (21%) reported they had not.

Regression analyses were conducted on 130 clinicians and team leaders across 26 teams (mean of five observations per cluster). Clinicians and team leader RSA scores were not associated with personal experience of mental illness (*b* = 0.09, 95% CI −0.07 to 0.24, *P* = 0.273) or supporting a family member or friend (*b* = 0.02, 95% CI −0.15 to 0.19, *P* = 0.836).

## Discussion

In this national survey across England, we compared variations between NHS trust, team type and participant ratings of recovery orientation of mental health teams. We identified influences on recovery orientation rating. The site (i.e. NHS trust) accounted for some variance, as did team type, with early psychosis teams having a higher recovery orientation than teams working with longer-term users of mental health services. Team leaders rated a greater recovery orientation than either clinicians or service users. Service users who rated a higher recovery orientation of the team also rated higher self-assessed recovery. Finally, no association was found in the clinician group between lived experience (either personal experience of mental illness or through supporting a family member or friend) and recovery-orientation rating.

### Predictors of recovery orientation

This study provides preliminary evidence that the overall recovery-orientation scores in English community-based mental health teams were high, with some recovery domains being very high (for example, life goals *v.* medication management) and others (for example, user involvement) lower. The RSA mean scores for team leaders were higher than those of service users and clinicians, whereas scores did not differ between clinicians and service users. Candidate reasons for higher team leader scores include social desirability (discussed later), overoptimistic or inaccurate appraisal of practice and different thresholds for recovery-oriented practice. By contrast, the RSA mean scores in a USA study were all higher than we found, and were highest for individuals in recovery (mean 4.06, *n* = 326) and directors (mean 4.09, *n* = 68), with providers (mean 3.89, *n* = 344) scoring the lowest.^5^ These variations, along with our finding of a site-level effect, highlight the need for larger-scale epidemiologically representative surveys using cross-culturally valid measures.

In the UK, community-based mental health teams serve different clinical populations and include both generic CMHTs and specialist mental health teams, such as early intervention and assertive outreach teams. The differences in recovery orientation between these teams may be as a result of different clinical populations (for example, proportion of people with psychosis or length of time using services) or team characteristics (for example, specialist workforce skills). A study of 67 assertive community treatment (ACT) teams in Canada found no relationship between ACT fidelity and recovery orientation, leading the authors to conclude that traditional fidelity measures may not adequately address the dimension of recovery-oriented service provision.^12^ However, integrating evidence-based recovery-oriented interventions into existing service models can be problematic, with difficulties such as fidelity, feasibility and acceptability reported.^13^ In relation to early psychosis teams, there may be a greater alignment between practice and the broader understanding of recovery held by first-episode patients^14^ than in teams providing longer-term care.

### Recovery orientation and recovery in service users

To our knowledge, this is the first empirical study that has found an association between service user perceptions of recovery orientation and their own personal recovery. Key recovery outcomes are connectedness (i.e. social inclusion), hope, a positive identity, meaning and purpose and empowerment.^15^ A moderate evidence base indicates that the relationship between these recovery outcomes and traditional clinical outcomes is weak. For example, recovery indicators are sensitive to stage of recovery whereas clinical outcomes are not,^16^ and functioning is not associated with recovery.^17^ Overall, psychosocial factors emerge as more influential on recovery than neuropsychiatric factors,^17^ which may have implications for the development of recovery-oriented service models.

### Recovery orientation and lived experience in clinicians

Over a third of clinicians and team leaders reported having experience of mental illness, less than half of whom had fully disclosed this experience to their workplace colleagues. Three-quarters had experience of providing informal support to a friend or relative with mental illness. An identified challenge for organisations intending to translate recovery rhetoric into practice is to transform and rebalance the skill-mix within their mental health workforce, with a much greater involvement of people with lived experience.^18^ This rebalancing of the skill-mix can be achieved partly through recruitment of service users to roles such as peer support workers and patient representatives, but also through clinicians with lived experience of mental illness. The existence within the workforce of a sizeable proportion of people with ‘dual identity’ of lived experience and professional expertise represents an untapped resource that may benefit others, for example by being more oriented towards strengths-based practice^19^ and the mental health system.^20^ There have been improvements in employers' mental-health-related knowledge, attitudes, and employment practices around recruiting and supporting employees with mental health problems. A recent series of surveys suggests that UK employers' are becoming less likely to perceive employing people with mental health problems as a risk with respect to their reliability or in terms of their colleagues' reactions to them.^21^ Organisations that successfully challenge within-system stigma^22^ are more successful at implementing the policy imperative of developing the peer specialist workforce.^23^ Therefore, mental health organisations may consider the benefits of actively valuing this lived experience within existing clinicians, and supporting clinicians to disclose this experience to colleagues and service users where wanted and appropriate.

### Strengths and limitations

The study had several strengths. The purposive sample from five English regions maximised variation in levels of urbanisation, deprivation and ethnicity. Participating NHS trusts differed in size and structure, again providing an ecologically valid perspective from routine service settings.

We also identify limitations. Although the teams were randomly selected from all community-based mental health teams within the NHS trust, the clinician sample was selected via convenience sampling. This may have led to a selection bias, for instance, with clinicians who strongly felt their practice was recovery-oriented being more likely to participate. Several professional groups were underrepresented in this survey (for example, psychiatrists and psychologists). Previous research has shown that professionals have different understandings of recovery.^24^ We found that clinicians and team leader recovery orientation of services scores were not associated with personal experience of mental illness or supporting a family member or friend. This may have been because of the relatively small sample size of those without lived experience and/or the diversity of illness and caring experiences potentially covered by these questions.

Of the 501 service users who were assessed for eligibility, many were not eligible for reasons such as no longer being on the case-load when researchers contacted them, or being uncontactable. However, access to service users was via clinicians and 45 service users were not asked by clinicians and 89 refused to participate, which may reduce representativeness. Additionally, only service users who were judged by the clinician to be sufficiently well to participate were included, and service users more advanced in their recovery may differ in their opinions from those earlier in their recovery. Interview respondents may have experienced more social desirability bias than those completing a questionnaire, although the interviewers were researchers and hence independent from the clinical team.

The QPR measure was developed using a UK mental health population who had experience of psychosis and was recommended in a recent systematic review,^25^ but has not been validated in a population of people with other mental illnesses. The RSA measure was developed in the USA and its cross-cultural validity has yet to be established. A systematic review of recovery support measures identified that some RSA items required service users to comment on service delivery, which they could not reasonably be expected to know about, given the way services are configured within the UK (for example, the cultural diversity clinician training item on the user involvement subscale).^26^ Given the international policy focus on providing recovery-oriented services,^27^ the RSA (especially team leader version) may be susceptible to social desirability bias. A social desirability scale to estimate the extent of this bias could have been included.

### Clinical and research implications

Despite the policy goal of increasing recovery orientation of mental health services, routine outcome monitoring of the recovery orientation of services is not common practice. Moving beyond the adoption of recovery principles through to persistent implementation of recovery-oriented practice into routine care entails putting effective feedback systems in place for both staff and policy-makers.^28^ To date, there has been little empirical UK-based evidence available for clinicians to gauge whether their work is recovery-oriented or help them reflect upon areas of practice they could target for service development. A study assessing the recovery orientation of 78 mental health and addiction programmes in Connecticut, USA, involved providing individual teams with structured feedback on their RSA total and subscale results to help them assess their own progress towards implementing recovery practice.^29^ When teams disseminated the findings of discrepancies between participant groups in the perception of recovery orientation, this led to service improvement. Studies comparing clinicians', carers' and service users' perceptions of need find differing perspectives, which in routine practice can lead to a shared commitment to provide more needs-led care.^30^ There is evidence that feedback of outcome data can improve the quality of mental healthcare^31^ and the routine use of new and psychometrically adequate measures of recovery support such as INSPIRE^32^ has been recommended for organisational transformation.^33^

This study has shown a cross-sectional association between recovery orientation in a mental health team and recovery experience of the service user. Criteria for demonstrating a causal relationship are association (defined as the putative cause and effect having temporal and spatial contiguity), direction (defined as cause precedes effect) and isolation (defined as the effects of a cause are isolated from other possible causes).^34^ Future research might investigate whether association and isolation are retained in longitudinal designs, and use a repeated measures design to test whether increasing recovery orientation leads to subsequent increase in recovery. Demonstrating that recovery support leads to improved recovery will further justify the development of recovery-oriented services.^35^
